# Advances in Zinc-Containing Bioactive Glasses: A Comprehensive Review

**DOI:** 10.3390/jfb15090258

**Published:** 2024-09-08

**Authors:** Fariborz Sharifianjazi, Mohammadjavad Sharifianjazi, Maryam Irandoost, Ketevan Tavamaishvili, Mehdi Mohabatkhah, Maziar Montazerian

**Affiliations:** 1 Center for Advanced Materials and Structures, School of Science and Technology, The University of Georgia, Tbilisi 0171, Georgia; 2 Department of Civil Engineering, School of Science and Technology, The University of Georgia, Tbilisi 0171, Georgia; 3 Department of Material Engineering, Isfahan University of Technology, Isfahan 84156-83111, Iran; mjsharifiancom877@gmail.com; 4 Department of Materials and Metallurgical Engineering, Amirkabir University of Technology, Tehran 15916-34311, Iran; mirandost@aut.ac.ir; 5 School of Medicine, Georgian American University, 10 Merab Aleksidze Street, Tbilisi 0160, Georgia; 6 Department of Engineering, Maku Branch, Islamic Azad University, Azerbaijan 58619-93548, Iran; mehdimohabatkhah@gmail.com; 7 Department of Materials Science and Engineering, The Pennsylvania State University, University Park, PA 16802, USA; mbm6420@psu.edu

**Keywords:** bioactive glasses, zinc, biomaterials, wound, tissue engineering

## Abstract

Bioactive glasses (BGs) have attracted significant attention in the biomaterials field due to their ability to promote soft and hard tissue regeneration and their potential for various clinical applications. BGs offer enriched features through the integration of different therapeutic inorganic ions within their composition. These ions can trigger specific responses in the body conducive to a battery of applications. For example, zinc, a vital trace element, plays a role in numerous physiological processes within the human body. By incorporating zinc, BGs can inhibit bacterial growth, exert anti-inflammatory effects, and modify bioactivity, promoting better integration with surrounding tissues when used in scaffolds for tissue regeneration. This article reviews recent developments in zinc-containing BGs (ZBGs), focusing on their synthesis, physicochemical, and biological properties. ZBGs represent a significant advancement in applications extending beyond bone regeneration. Overall, their biological roles hold promise for various applications, such as bone tissue engineering, wound healing, and biomedical coatings. Ongoing research continues to explore the potential benefits of ZBGs and to optimize their properties for diverse clinical applications.

## 1. Introduction

The newly proposed definition of bioactive glass is “a non-equilibrium, non-crystalline material designed to induce specific biological activity”. Bioactive glasses (BGs) have the capability to bond with bone and soft tissues or aid in their regeneration, making them valuable in applications such as orthopedics, dentistry, and wound repair. Additionally, they can be used to deliver specific concentrations of inorganic therapeutic ions, provide heat for magnetic-induced hyperthermia or laser-induced phototherapy, emit radiation for brachytherapy, and facilitate drug delivery to combat pathogens and cancers [1,2,3,4]. Larry Hench pioneered their development in 1969, introducing the initial formulation known as 45S5 Bioglass^®^ [5,6]. This particular composition not only establishes a connection with the bone but also stimulates bone cell development and repair. Its applications have extended beyond dental and bone defect treatment, encompassing soft tissue regeneration, orthopedic, nerve renewal, and drug delivery [7,8,9,10,11,12,13]. Metallic elements such as iron, magnesium, cobalt, and zinc are used to modify BGs’ performance [14,15,16,17]. The literature database reveals a growing number of publications exploring the impact of these ions on bone tissue repair and regeneration, demonstrating a notable increase in interest in this field of study [18,19,20,21,22,23,24,25]. This underscores the potential of incorporating dopants into BGs to enhance bone regeneration and tissue integration. Zinc plays a vital role in multiple facets of human health, including genetic regulation, biochemical processes, immune function, cell signaling, DNA and protein synthesis, wound healing, growth promotion, and overall immunological defense alongside skin health maintenance [26,27,28,29]. Additionally, it should be mentioned that zinc exhibits antibacterial properties and has been found to possess osteo- and angiogenetic capabilities. Moreover, it has been observed to stimulate the proliferation of osteoblastic cells [30]. Inadequate zinc consumption can lead to adverse effects such as impaired growth, weakened immune system, and skin-related issues. By ensuring an appropriate level of zinc consumption via dietary sources or supplements, one can mitigate these risks and preserve optimal bodily functions. Innovative developments centered around zinc-infused BG have attracted substantial scientific interest because they hold great potential in areas like orthopedics [31], dentistry, and wound healing [32,33]. Extensive research has been conducted to comprehend the physical, mechanical, biological, and structural properties of these glasses, along with assessments of both laboratory (in vitro) and animal model (in vivo) behaviors. Notably, zinc has been observed to boost BG bioactivity and unique physical, mechanical, and structural characteristics. This discovery opens up exciting possibilities for advancing medical technologies and improving patient care across various therapeutic domains [34,35].

Zinc plays a crucial role in the mineralization, creation, growth, and preservation of strong bones, making it a desirable component for usage in bone tissue engineering [34]. BGs enriched with up to 5 mol% ZnO have been assessed for their ability to promote bone regeneration, with encouraging outcomes [36]. The introduction of zinc, in combination with elements like manganese, copper, strontium, and magnesium, into BGs has been proven to influence the bioactivity of the base glass, paving the way for the advancement of innovative BG materials [37,38]. For example, the incorporation of zinc into BGs not only enhances antibacterial properties but also stimulates the differentiation and proliferation of bone cells related to osteoinductive and osteoconductive properties [37,39,40].

Research on ZBGs shows promise for various usages within the domain of bone regeneration and soft tissue repair [41,42]. The expanding research on BGs containing zinc emphasizes their potential significance in the field of biomedicine [43,44,45]. This review article explores the impact of zinc-doped BGs on various biological functions, including angiogenesis, osteogenesis, antibacterial properties, and its potential in biomedical applications. Furthermore, it explores their possible uses in bone tissue engineering, such as scaffolds, bone fillers, grafts, and coatings [46].

## 2. Synthesis of Zinc-Doped BGs

**Figure 1:**
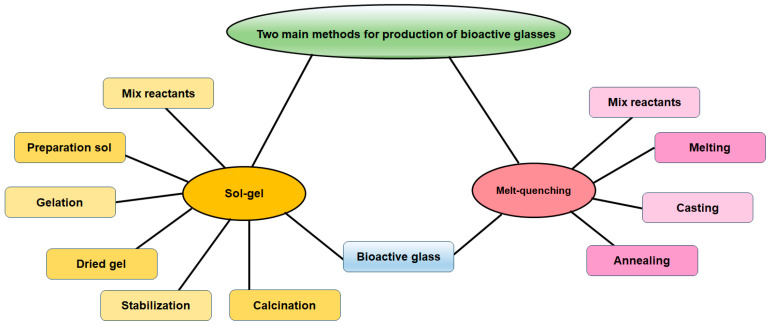
Main methods for synthesis of bioactive glass.

There exist two primary categories of manufacturing methods utilized in BG manufacturing: melt-quenching processes and sol-gel techniques (Figure 1). This section briefly discusses melt-quenching and sol-gel BGs, emphasizing their technological features and overall properties. Both techniques result in the creation of bioactive materials, although certain compositional constraints must be taken into account specifically for melt-quenching BGs [47,48]. The process of melt-quenching necessitates the careful adjustment of the composition to enable smooth melting and shaping (powder, monolith, fiber, thin film, etc.) that are tailored to the desired final application. Additionally, this adjustment ensures that the composition meets the necessary criteria for reactions in biological fluids. In this stage, a precise assessment of the existing technologies for implementing the process should be conducted. Conversely, the sol-gel technique enables the broadening of the compositional range while maintaining the system’s bioactivity and overcoming certain constraints associated with melt-derived glasses. For instance, issues related to undesired reactions with crucibles due to the presence of metallic ions and phosphorous are non-existent. Additionally, the sol-gel method allows for lower processing temperatures. This is because calcination temperatures are lower than melting temperatures, which also facilitates the preparation of powders with high surface area and porosity [47,49].

### 2.1. Melt-Quenching

Before the 1990s, the manufacturing process of BGs predominantly relied on melting techniques, with a significant focus on research studies centered around the 45S5 BG. Melting methods entailed the fusion of oxides and additives at elevated temperatures, followed by fast-quenching of the melt and then grinding the glass particles into fine powder [50]. The components undergo a melting process at high temperatures (usually within the range of 1200 °C to 1550 °C) using electric furnaces. The parameters are carefully adjusted to ensure a homogenous melt. At this stage, the temperature is selected to ensure that the viscosity of the molten material is less than 100 Poise, thus aiding in the removal of bubbles and further homogenization [51]. The melting time (t_m_) in laboratory experimental practice typically ranges from 1 h to 24 h, depending on the batch size [51]. The melting procedure can be repeated multiple times to achieve extremely high levels of uniformity. The forming routes that can be utilized depend on the desired final shape, and include the following options:

- The process of shaping through casting in molds, rapid cooling in water, or drawing into continuous fibers.
- Heating the glass above its glass transition temperature (T_g_) enables the particles to sinter, forming a porous scaffold, allowing for the drawing of fibers from a pre-form, or facilitating the sealing of particles to create coatings on a surface [52].

The melt-quenching method has been employed to make glasses with specific particle sizes after milling. Currently, the predominant commercial method for producing bioactive glasses involves the fabrication of over 99% of them through melting. Melt-quenching also allows for changing compositional space and enables the incorporation of transition metals and rare earth elements through doping [53]. The challenges encountered in this approach involved the elevated operating temperature and the vaporization of some components like Na_2_O, P_2_O_5_, etc. [54,55].

### 2.2. Sol-Gel

The sol-gel process is a widely used chemical synthesis method for producing inorganic materials, such as ceramics, glasses, and metal oxides, with unique properties and microstructures. Employing the sol-gel method enables the production of BG nanoparticles (BGN) with enhanced controllable morphology and size, as the precursors are mixed and permitted to undergo a reaction in a liquid environment [56,57,58]. Sol-gel processing offers numerous benefits compared to conventional melt-quenching techniques for BGs. These advantages include increased homogeneities and purities, reduced operation temperatures, wider varieties of bioactive structures, higher specific surface area, accelerated apatite layer formation, and quicker bone regeneration, as well as improved degradability and in vivo resorption [59]. BGs produced via the sol-gel technique have demonstrated a minimal inflammatory response [60] and can enhance the bone regenerating process [61]. Furthermore, the sol-gel method enables the convenient integration of various inorganic ions into the composition of the glass [62]. The sol-gel technique is a wet-chemical procedure utilized to fabricate materials by using components of metallic ions, silicate, phosphate, and borate, particularly for BGs [63]. The procedure primarily consists of the hydrolysis and condensation of substances, followed by drying and stabilization. Via regulating the operating criteria, it is possible to manipulate the characteristics of materials, including morphology and composition [64]. The most commonly utilized silicate precursor for the production of sol-gel BGs is tetraethyl orthosilicate (TEOS), with water and/or ethanol serving as the preferred solvents in the process [47]. The sol-gel method is capable of occurring in either acidic or alkaline environments, and these varying conditions have an impact on the characteristics of the materials produced. By adjusting the pH level of the solution, it is possible to obtain BGs with diverse structures. In a standard sol-gel procedure, TEOS initially experiences hydrolysis and condensation with catalyst assistance to generate [SiO_4_]^4−^ structural units [64]. Metallic ions may be introduced or doped either in the initial stages of TEOS condensation or following the diffusion into a SiO_2_ structure after drying and calcination processes to yield BGs. Throughout the production process, organic compounds may be incorporated to control the morphology of the particles or enhance their dispersion [65]. Furthermore, the sol-gel method has been extensively studied in recent years as a viable alternative to the melting process. Additionally, the incorporation of other techniques, such as microemulsion [66], can enhance BGs production via the sol-gel procedure [67]. Alongside the regular melt-quenching method, the sol-gel approach offers an alternative means of synthesizing BG as indicated in Table 1.

## 3. Physico-Mechanical Properties of ZBGs

Alterations in the mechanical and physical properties of BGs after incorporating specific ions have a notable influence on both in vivo and in vitro biological responses, as well as interactions with ions released from the glass [75]. Zinc has been associated with changing the melting point [76,77]. Nicolas Rocton et al. [78] conducted a research study to examine the impact of Zn on the thermal properties of glass. The study focused on investigating crystallization temperature (T_c_), melting temperature (T_f_), and glass transition temperature (T_g_). Additionally, researchers calculated the thermal stability (T_s_) and excess entropy of all the glasses under investigation. Notably, the melting temperature of the glass decreased significantly when doped with 10 wt% Zn. Moreover, the addition of Zn resulted in a decrease in the excess entropy. With regards to the impact on thermal properties, the addition of Zn to the SiO_2_–CaO–Na_2_O–P_2_O_5_ system typically results in a reduction in the melting temperature. Additionally, the presence of Zn causes the glass transition temperature to drop [79]. In the investigation conducted by Mohan Babu et al. [80], the focus was on studying the improved physico-mechanical properties of ZnO-doped phosphate BGs. A rise in ZnO concentration led to an increase in physical parameters such as oxygen molar volume and density, while oxygen packing density decreased.

In recent years, studies have concentrated on incorporating modifier ions into BGs to enhance their therapeutic efficacy. The ability to adjust the glass composition to include various elements in varying amounts has led to improvements in their physical and chemical characteristics, making them more suitable for particular uses [81]. ZnO can modify the network structure of 80SiO_2_–15CaO–5P_2_O_5_ in mol% (80S) glass. ZnO can function as either a glass former or a network modifier, depending on the concentration (x) in the glass system. When present in low amounts, ZnO functions as a network modifier, as evidenced by Yu-Hsuan Chen et al. [82]. It has been confirmed that adding ZnO to the 80S glass results in the zinc ions (Zn^2+^) forming tetrahedral [ZnO_4_]^2−^ coordination as network formers. This causes the calcium ions (Ca^2+^) to behave as charge compensators, leading to the locations of both Ca and Zn being nearby within the non-bridging oxygens.

**Figure 2:**
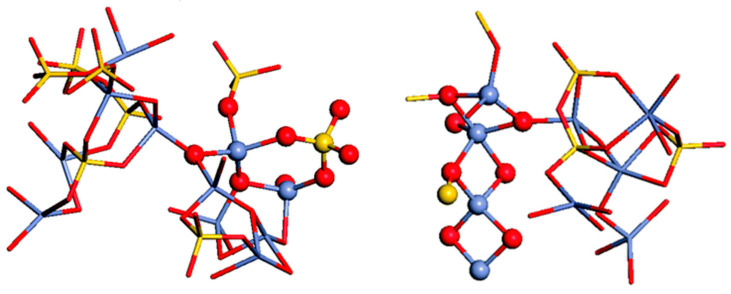
The [ZnO_4_] and [SiO_4_] network, highlighting the edge-shared Zn–Si and Zn–Zn polyhedra. Oxygen atoms are represented by red balls, silicon atoms by yellow balls, and zinc atoms by blue balls, reprinted with permission from Ref. [88]. Copyright 2013 Royal Society of Chemistry.

Numerous molecular dynamic (MD) investigations have been conducted to study the Zn effect in Bioglass-45S5-based glasses. The objective of these studies was to study glasses that maintain their bioactivity while exhibiting an ideal rate of zinc release. They have concluded that Zn plays an intermediate role. The majority of Zn ions (over 80%) are 4-coordinated and connect with the SiO_4_ tetrahedra. Also, a 5-coordinated Zn ion is found in the glasses [83,84,85]. These studies could contribute to glass design and achieve utilization of the quantitative structure–property relationships procedure [86]. One potential reason for this phenomenon could be the replacement of CaO with ZnO and MgO, causing structural modification and changes in the calcium content that may influence apatite formation [87]. Visualization allows for a direct examination of polyhedral structures and their interconnections, as depicted in Figure 2. The network of the glass is formed by standard [SiO_4_] tetrahedra and slightly distorted [ZnO_4_] tetrahedra. These polyhedra are predominantly connected by corner-sharing oxygen atoms. However, some [ZnO_4_] tetrahedra are linked to each other or to [SiO_4_] tetrahedra through two shared oxygens. Examples of these edge-shared polyhedra are presented in Figure 2. It was noted that edge-sharing frequently occurs between 5-coordinated zinc atoms, which are associated with 3-fold coordinated oxygen atoms.

The addition of zinc in concentrations exceeding 5 wt.% to silicate glass enhances its compressive strength and its resistance to aqueous environments [89]. This triggers multiple responses in the surface reactivity of bioactive glass and its interaction with bone cells, in addition to its antibacterial efficacy [90]. Ainaa et al. [91] emphasized the significance of developing Zn-containing 45S5 BG and supported additional knowledge into the pre-oxidant function of Zn in biological systems. Their research involved incorporating ZnO at concentrations ranging from 5% to 20% (*w*/*w*) in the glass. The researchers concluded that the elevated Zn content hindered its solubility by releasing Zn^2+^ ions into the solution, thereby disrupting metabolic processes. Then, Fandzloch et al. [92] presented their findings on two nano glasses with particle sizes equal to or less than 123 nm. These nano glasses were synthesized using the sol-gel method in a ternary (75SiO_2_−8CaO–17ZnO mol%) and binary (83SiO_2_−17CaO mol%) system. They successfully synthesized amorphous glass with a high zinc oxide content. The inclusion of zinc oxide in the glass composition was found to have an impact on its textural characteristics, such as surface area and pore volume, as well as its physiological properties, including dissolution characteristics and apatite forming capability. However, it is noteworthy that the nano glass 75SiO_2_–8CaO–17ZnO did not compromise biocompatibility, as demonstrated by its compatibility with HDF and MC3T3 cells.

## 4. Biological Roles of ZBGs

**Figure 3:**
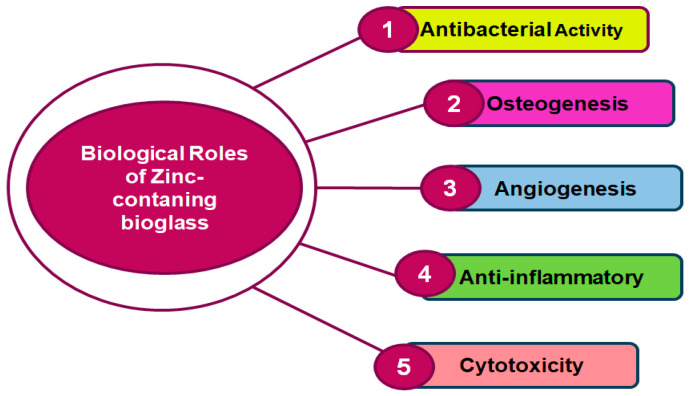
Biological roles of zinc-containing BGs.

ZBGs are well-known for their essential role in various biological applications, particularly in tissue regeneration and bone formation. Zinc, a vital mineral, is essential to the development of blood vessels, bone growth, and protection against bacteria [93]. When integrated into BG, zinc can enhance antibacterial and anti-inflammatory properties [42,94]. Figure 3 shows the biological roles of ZBGs. While zinc is essential for various biological processes, excessive levels can be cytotoxic. It is important to balance zinc release from BGs to ensure that it promotes beneficial effects without reaching toxic concentrations. Controlled release ensures that zinc supports cellular activities without causing harm to the surrounding tissues. These roles highlight the multifaceted benefits of incorporating zinc into BGs for biomedical applications, particularly in bone regeneration and tissue engineering. It enhances the antibacterial, osteogenic, and angiogenic properties of BGs, while also providing anti-inflammatory benefits. However, careful control of zinc release is crucial to avoid cytotoxic effects [95].

### 4.1. Antibacterial Activity

As noted before, zinc is important for the growth, generation, and function of bone cells, as well as in the development of blood vessels and its antimicrobial properties [96,97,98]. Additionally, it enhances the process of wound healing [99]. Zinc exhibits antibacterial properties by hindering transmembrane proton translocation, reducing glycolysis acid tolerance, and disrupting processes within bacterial cells [100]. Zinc ions are becoming more popular in bone regeneration applications due to their significant influence on bone formation and growth, as well as their antibacterial properties. This has led to an increasing interest in combining zinc ions with biomaterials for enhanced bone regeneration outcomes [101].

In a recent investigation [102], it was observed that the incorporation of 4 mol% ZnO in place of SiO_2_ in 80SiO_2_–15CaO–5P_2_O_5_ (mol%) BG scaffolds led to a notable increase in the quantity of deceased bacterial cells (*S. aureus*). Furthermore, the presence of this quantity of additional ZnO resulted in enhanced osteoblast growth following the immersion of zinc-containing scaffolds in culture medium extracts for durations of 1, 3, and 6 days.

**Figure 4:**
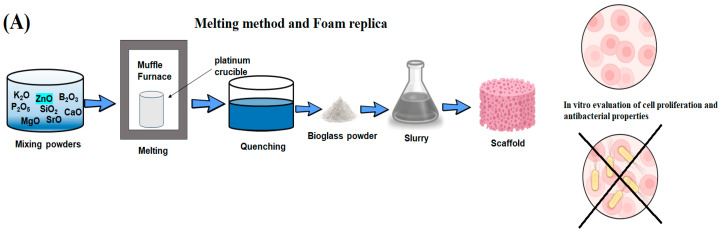

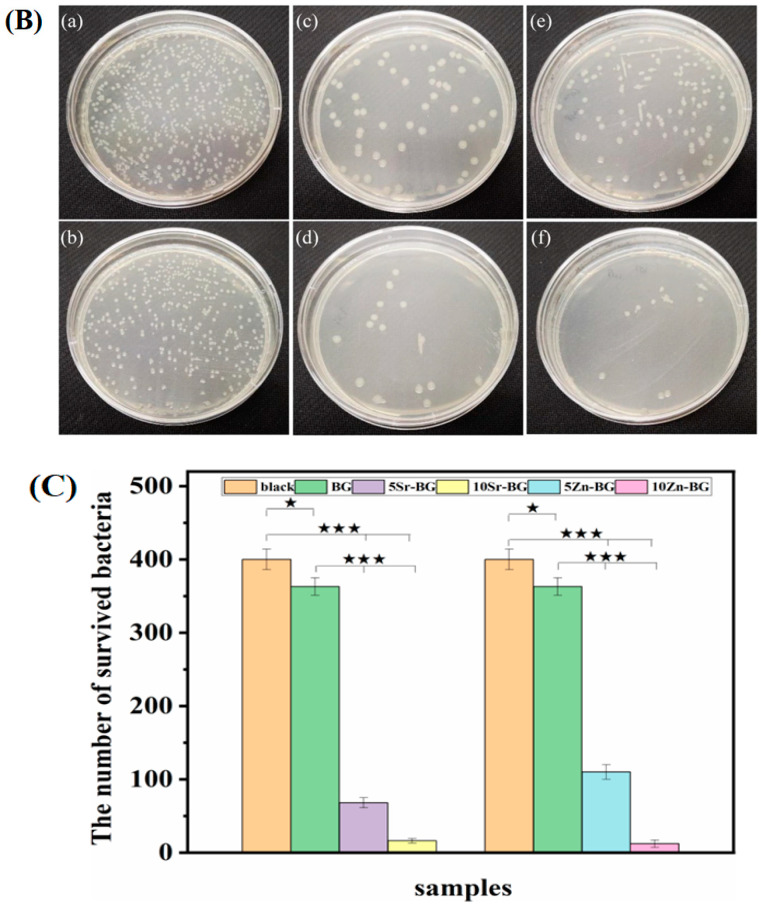
(**A**) Preparation of bioactive glass scaffolds. (**B**) Various images depicting *E. coli* bacteria cultivated in different conditions, including (**a**) a control group, (**b**) BG medium, (**c**) 5Sr-BG medium, (**d**) 10Sr-BG medium, (**e**) 5Zn-BG medium, in addition to the (**f**) 10Zn-BG medium [105]. (**C**) BG granules’ antibacterial efficacy against strains of *E. coli* bacterium represented through histograms (Differences were considered to be statistically significant for *p* < 0.05 (*) and *p* < 0.001 (***), reprinted from Ref. [105].

The influence of incorporating ZnO on the bioactivity and the silicate BGs’ biocompatibility with compositions similar to 58S BG (58SiO_2_–33CaO–9P_2_O_5_, wt.%) in addition to the 64S BG (64SiO_2_–26CaO–10P_2_O_5_, wt.%) has been investigated separately by Balamurugam et al. [103] and Bini et al. [104], respectively. They concluded that incorporating zinc into these BGs does not diminish their ability to form apatite after exposure to simulated body fluid (SBF). The addition of zinc is beneficial for cell attachment and helps maintain the pH within physiological limits. In vitro biocompatibility assessments indicate that substituting limited amounts of zinc in the BGs stimulates cell proliferation and promotes differentiation [103,104]. In a recent work carried out by Sanchez-Salcedo et al. [67], it was found that for the 80SiO_2_–15CaO–5P_2_O_5_ (mol%) meso-microporous scaffolds, adding 7% ZnO exhibited favorable biocompatibility and demonstrated effective antimicrobial activity against *S. aureus*. Rui et al. [105] prepared bioactive glass scaffolds containing ZnO or SrO (0, 5, and 10 mol%) using the melting and foam replica technique. They demonstrated that the proliferation of *E. coli* was inhibited by the BGs. The number of CFUs for 10Zn-BG (12 ± 5) and 10Sr-BG (16 ± 3) was significantly lower compared to 5Zn-BG (110 ± 10), 5Sr-BG (68 ± 7), BG (363 ± 12), and the control group (400 ± 14), as shown in Figure 4B,C. The Zn series and Sr series glasses exhibited a noticeable bactericidal impact. The bactericidal effectiveness of BG was found to be statistically significant comparing the control group (* *p* < 0.05). Furthermore, 5Zn-BG, 5Sr-BG, 10Sr-BG, and 10Zn-BG displayed an elevated level of antibacterial activity comparing the BG and the control group (*** *p* < 0.001). Essentially, the Sr- and Zn-doped samples’ antibacterial efficacy against the *E. coli* (ATCC 25922) bacterium exhibited a dose-dependent relationship [105].

### 4.2. Osteogenesis

**Figure 5:**
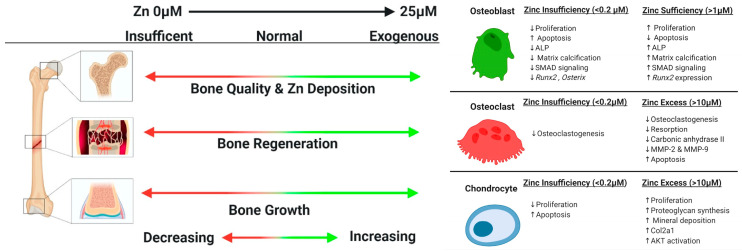
(**left**) Roles of zinc in the skeleton and (**right**) zinc effects on skeletal cells, reprinted from Ref. [112].

Zinc is a metallic ion that is anticipated to have a major effect on bone repair. In addition to its involvement in bone resorption, zinc also maintains bone mass and may be used as a therapeutic option for osteoporosis. The effect by which zinc functions to promote bone development is illustrated in Figure 5.

Zinc compounds have the potential to enhance the manufacture of bone matrix proteins and bone growth factors. These substances play a crucial role in promoting bone building in addition to stimulating cell proliferation in osteoblastic cells. Also, zinc is known to have a positive impact on bone health as it enhances mineralization and bone formation through the aminoacyl-tRNA synthetase activation in osteoblastic cells. Additionally, it promotes cellular protein synthesis. Furthermore, zinc aids in maintaining bone mass by preventing the formation of osteoclast-like cells from marrow cells. This can facilitate osteoblast connection and proliferation, leading to an increase in alkaline phosphatase (ALP) expression, which is crucial for bone callus formation. Zinc incorporation into bioactive glasses results in improved chemical stability in addition to the glass matrix densification. When zinc is doped into mesoporous BG nanoparticles (MBGNs) of 70SiO_2_–30CaO (mol%) composition for diaphyseal defect repair, it fosters a strong interaction between ZBG and bone in the initial stages, while in the later stages, there is resorption, osseointegration, in addition to the bioactive glass degradation, followed by its replacement with bone cells. In comparison to all the other MBGN groups and control group, the Zn-doped MBGN IDPs exhibited a significant upregulation of ALP activity, as reported by Fabian et al. [106]. The ALP activity exhibited a notable increase when exposed to IDPs of Zn-doped MBGNs. This increase was observed not only in comparison to the control group but also when comparing it to all other MBGN groups. The corresponding findings were published by Oh et al. [107] after cultivating rodent BMSCs using sol-gel-produced zinc-containing 70SiO_2_–30CaO (mol%) BGs for a duration of 14 and 21 days in culture. In controlling ALP function, Zn also exerts its influence. Yamaguchi and colleagues reported in 1986 that Zn not only triggers ALP activity but also boosts DNA synthesis, leading to the promotion of tissue-grown bone obtained from rodent femora [108]. Additionally, zinc is essential in aiding the activity in addition to the ALP functionality [101]. The gene expression of OPN and OCN was found to be upregulated in IDPs of 5Zn-MBGNs in comparison to both the undoped MBGN IDPs and the control group. Additionally, it was observed that 5Zn-MBGN IDPs caused a notable rise in collagen synthesis in addition to extracellular matrix (ECM) calcification in comparison to the control group or undoped MBGNs. ECM mineralization downregulation due to Zn deficiency is associated with decreased ALP function in osteoblasts, highlighting the crucial role of Zn in ECM mineralization [101]. Huang et al. [109] illustrated that the introduction of human dental pulp stem cells to inorganic polyphosphate compounds found in ZBGs resulted in elevated mineralized nodule levels. Courthéoux et al. [62] demonstrated the enhancement of the initial stage of the bioactive process through the incorporation of zinc as a dopant. Zinc plays a crucial role in increasing the specific surface area, thereby promoting the nucleation site formation for calcium phosphate precipitates. Moreover, adding zinc decelerates glass dissolution and facilitates the growth of the calcium phosphate layer on the ZnO–SiO_2_–CaO glass surface. This process can be essential for the successful fusion of BGs to living bone tissues [62]. In the continuation of roles of zinc-containing bioactive glass, Saino et al. [110] examined the Zn-doped 58S BG effects on Saos-2 cells. Their findings revealed both a notable rise in ALP function and a significant augmentation in calcium deposits within the groups that were cultured in the presence of Zn-doped BG. Hence, 5Zn-MBGNs exhibit the highest advantageous pro-osteogenic characteristics within the current context, making them potential contenders for future implementation in bone tissue engineering (BTE). This could include their utilization in in vivo models, for instance. Zheng et al. [111] reported the zinc-modified BG osteogenic impact on mesenchymal stem cells, even at a concentration as low as 0.95 mg L^−1^.

### 4.3. Angiogenesis

Angiogenesis plays a crucial role in tissue regeneration by facilitating the delivery of oxygen and nutrients to the newly formed tissue. Consequently, the impact of BG on endothelial cells and their vascularization has garnered significant interest and has been extensively researched. Zinc plays a crucial role as an element in numerous proteins and is pivotal in a multitude of biological processes [113]. Also, the release of zinc ions was essential in stimulating soft tissue wound healing via increasing endothelial cell angiogenesis. Furthermore, the liberation of zinc ions is important for promoting the soft tissue wound healing process through the facilitation of angiogenesis in endothelial cells. Numerous elements are involved in the angiogenesis process, such as growth factors like extracellular matrix proteins and vascular endothelial growth factor (VEGF), in addition to the membrane receptor quantity and nature. Also, specific compositions of BGs have the potential to induce fibroblasts to release proteins and growth factors, like fibronectin, type I collagen, vascular endothelial growth factor (VEGF), basic fibroblast growth factor (bFGF), and epidermal growth factor (EGF), which could aid in enhancing wound healing through multiple mechanisms, such as heightened vascularization [114]. Angiogenesis is important in the wound healing mechanism by facilitating the re-establishment of proper blood circulation, the delivery of sufficient nutrients and oxygen, and the removal of metabolic byproducts, which are vital for cell growth and survival. A loosely structured temporary extracellular matrix is indicative of strong angiogenesis in a properly healing wound, resulting in its recognizable swollen and pink look [115]. Zhang et al. [116] have shown that the incorporation of ZBG into α-tricalcium-phosphate-based cements (CPCs) can activate odontogenic differentiation in addition to in vitro angiogenesis stimulation by utilizing the NF-κB, MAPK, Wnt, and integrin pathways. This suggests that ZnBGs with CPCs have the potential to be effective matrices in tissue engineering for enhancing endodontic regeneration [116].

Studies focusing on zinc metalloenzymes, like alkaline phosphatase, have provided further evidence of the important role of zinc in regenerating mechanisms. Alkaline phosphatase is particularly significant as it acts as a delicate indicator for fine dermal blood vessels and the angiogenesis initial phases, which are linked to heightened inflammatory responses and the proliferation of connective tissue. Additionally, DNA polymerases have been identified as reliable markers for cell proliferation [117].

### 4.4. Anti-Inflammatory

Zinc ions exhibit anti-inflammatory, antibacterial, and anti-gingivitis properties. When incorporated into BGs, zinc enhances its versatility as a material for dental restoration and bone augmentation [101,118]. The presence of zinc ions is advantageous in various stages of wound healing due to their antimicrobial effects, particularly in the initial phases of healing. Additionally, zinc ions possess anti-inflammatory properties that help reduce oxidative stress and inflammatory cytokines. Moreover, they stimulate neo-angiogenesis, playing a crucial role in both the proliferation and remodeling phases of wound healing [119]. The impact of bioactive compounds on immune cells, as a crucial element in controlling the immune response, is currently under scrutiny. Numerous immune cells participate in the immune response. Among these cells, macrophages have become a principle focus due to their pivotal functions in overseeing various stages of tissue repair and regeneration [120].

**Figure 6:**
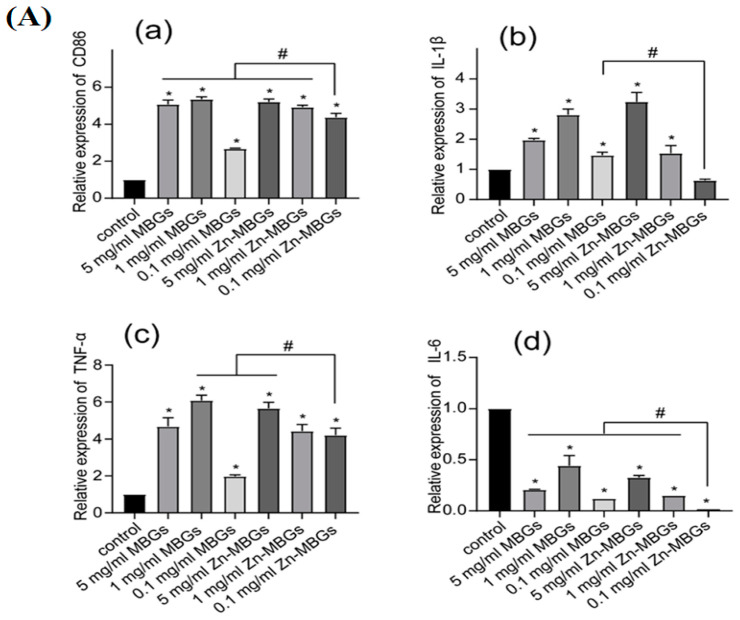

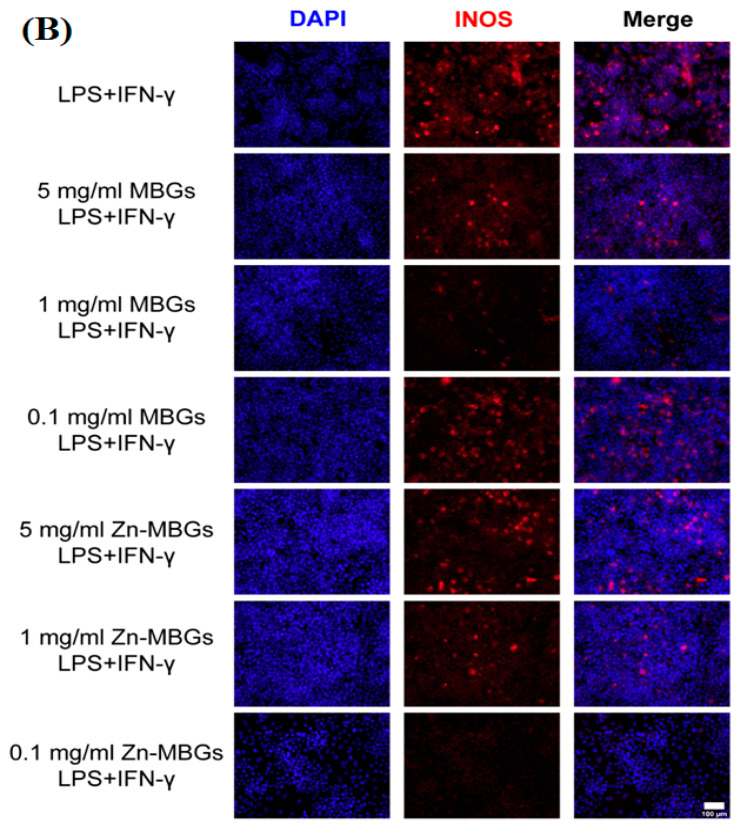
(**A**) The mRNA expression levels of inflammatory cytokines, namely CD86 (**a**), IL-1 β (**b**), TNF-α (**c**), and IL-6 (**d**), were assessed in RAW264.7 cells treated with Zn-MBGs in addition to MBGs. (Significant difference between the intervention and control groups with * *p* < 0.05; significant difference between groups with # *p* < 0.05.) (**B**) Fluorescent INOS staining was performed on RAW264.7 cells treated with LPSand IFN-γ following a 24 h culture with MBG and Zn-MBG extracts, reprinted from Ref. [44].

The study by Haishui Sun et al. [44] delved into the impact of Zn-MBGs on macrophage polarization. RAW264.7 cells were treated with varying concentrations of particle extracts for a duration of 24 h, following which an assessment of cell phenotypes and inflammatory factor expression was conducted. RAW264.7 cells possess distinct macrophage properties and can transition into different cell phenotypes under the influence of diverse stimuli. The analysis presented in Figure 6 reveals a significant upregulation of the cell membrane protein CD86 (a crucial M1 macrophage marker) via the extracts, regardless of their concentrations. This indicates that the presence of MBGs and Zn-MBGs can induce macrophages to adopt a pro-inflammatory M1 phenotype. The pro-inflammatory mediators TNF-α and IL-1β were also upregulated simultaneously, signifying the inflammatory response initiation provoked by Zn-MBGs and MBGs. Particularly noteworthy is that Zn-MBGs, especially at lower concentrations (0.1 and 1 mg/mL), exhibited a better ability to downregulate pro-inflammatory IL-1β and IL-6 expression when compared to MBGs. Also, to assess the Zn-MBG anti-inflammatory efficacy, an immunofluorescence study was conducted to evaluate the induction of inducible nitric oxide (INOS), a key enzyme involved in the inflammatory response. Notably, Zn-MBGs at a 0.1 mg/mL concentration exhibited the lowest level of INOS induction compared to other groups. This was confirmed by the weakest red fluorescence observed, thereby confirming the considerable Zn-MBG anti-inflammatory effects at this specific concentration. Bosetti et al. [121] conducted a study to explore the interaction between different types of glasses, such as Ag-doped phosphate BGs, Zn-doped phosphate BGs, and crystalline quartz powders, 45S5 BG, with macrophages. The aim was to investigate these glasses’ potential anti-inflammatory properties. The researchers found that when an inflammatory stimulus (LPS) was present, 45S5 BG significantly decreased the pro-inflammatory cytokines TNF-α and IL-6 secretion in macrophages compared with the group without 45S5 BG. Additionally, Zn-doped BGs also exhibited a reduction in IL-6 secretion. Interestingly, the presence of all BGs led to an increase in IL-10 secretion, an anti-inflammatory cytokine. Another study indicated that Zn has the ability to prompt monocytes to transform into macrophages; however, the orientation of macrophages’ polarization is contingent upon the level of Zn present. For instance, Zn^2+^ at a concentration of 100 × 10^−6^ M has been shown to enhance the release of pro-inflammatory cytokines (IL-6, TNF-α, and IL-1β). Conversely, Zn^2+^ at a 1.25 × 10^−6^ M lower concentration was found to diminish the pro-inflammatory cytokine expression [122,123]. Varmette et al. [124] explored the Cu- and Zn-doped sol-gel BG effects on macrophages. The results indicated that 58S BG exhibited a reduction in the expression of the pro-inflammatory gene TNF-α in macrophages. Additionally, the study found that Cu and Zn incorporation in 58S BG led to greater suppression of pro-inflammatory genes, underscoring the anti-inflammatory properties of Zn- and Cu-doped BGs.

### 4.5. Cytotoxicity

Zinc has the potential to trigger cytotoxic effects in cells as its concentration escalates to the high nanomolar to micromolar range, resulting in observable alterations in cellular and mitochondrial structure. Research has demonstrated that an excess of zinc can instigate stress in both cellular and mitochondrial environments, impacting mitochondrial structure and prompting cytotoxicity. Furthermore, studies have identified that zinc oxide nanoparticles possess cytotoxic and genotoxic properties, which are closely linked to the production of reactive oxygen species (ROS). The cytotoxic impact of zinc is associated with its capacity to elevate oxidative stress levels, ultimately leading to cellular harm and demise. Hence, it is imperative to meticulously control zinc levels to avert its cytotoxic repercussions within the body. In 2007, Aina et al. [91], conducted a study on the cytotoxic effects of Zn-doped BGs, examining the impact of Zn dopant concentration on endothelial cells in addition to human osteoblasts. Their findings revealed that Zn concentrations ranging from 2 to 8 ppm could induce harm in human osteoblasts through oxidative stress. Moreover, they observed that BGs doped with 20 wt.% Zn (20Zn-BG) enhanced endothelial cell adhesion but reduced their proliferation rate. Conversely, BGs doped with 5 wt.% Zn (5Zn-BG) promoted both cell adhesion and proliferation.

**Figure 7:**
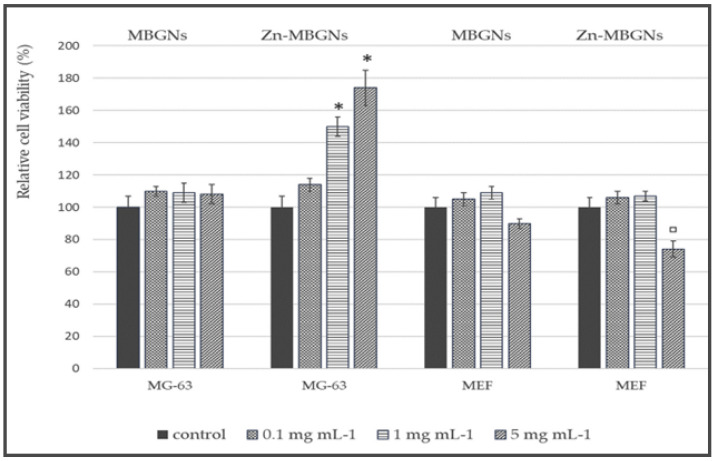
The quantification of the amount of viable MEF and MG-63 cells in a cytotoxicity assay was conducted using elution extracts of MBGNs and Zn-MBGNs at concentrations of 0.1 mg mL^−1^, 1 mg mL^−1^, and 5 mg mL^−1^. (* (*p* < 0.05) significant increase respect to the control; ▫ (*p* < 0.05) significant decrease respect to the control), reprinted from Ref. [43].

Additionally, Neščáková et al. [43] investigated BG particles (70SiO_2_–30CaO, in mol%) that were either undoped or doped with Zn (Zn-BG) through the utilization of the microemulsion-assisted sol-gel technique. The samples, whether doped or undoped, exhibited a spherical morphology with a particle size measuring 130 ± 10 nm. Evaluation of in vitro bioactivity through the use of SBF solution indicated that Zn inclusion had the effect of retarding hydroxycarbonate apatite generation on the surface of the particles. Furthermore, a consistent and prolonged Zn^2+^ release was detected. Moreover, Zn-BG exhibited no cytotoxic effects on osteoblast-like cells in addition to the embryonic fibroblasts, thereby promoting these cell types’ proliferation. Furthermore, the presence of Zn-BG resulted in enhanced protein adsorption compared to undoped BG, as depicted in Figure 7.

Haimi et al. [125] and Aina et al. [126] have both examined the potential toxic impact of zinc on cells in their respective studies. The addition of approximately 5 mol% was regarded as the threshold concentration of zinc, beyond which no cytotoxic effects were observed [126,127,128]. The elution extracts derived from Zn-MBGNs demonstrated detrimental impacts solely on the MEF cell proliferation when administered at the 5 mg mL^−1^ maximum concentration.

## 5. Biomedical Applications of ZBGs

### 5.1. Bone Tissue Scaffolds

**Figure 8:**
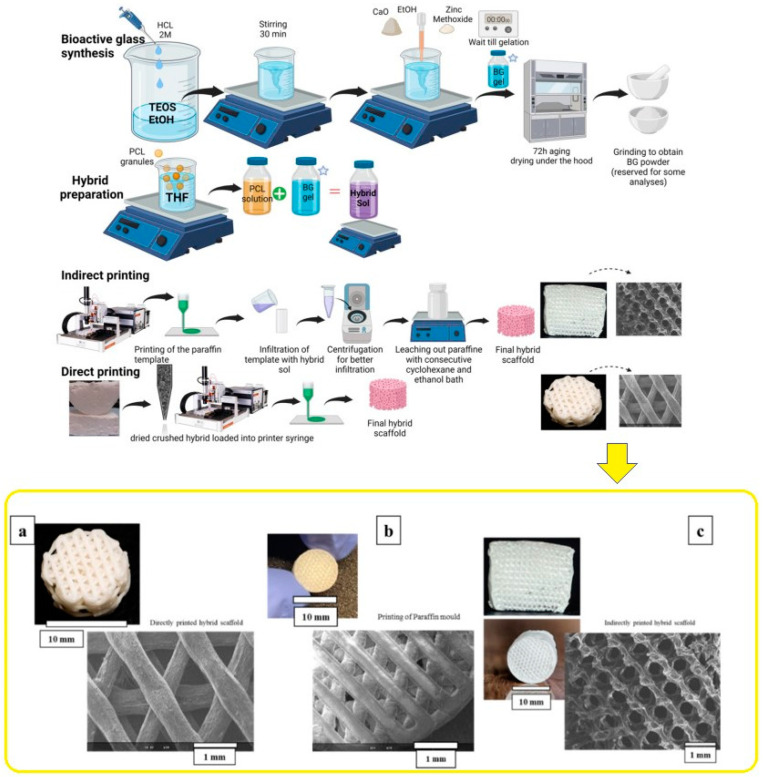
Schematic of scaffold processing and SEM showing the formation of hybrid scaffolds through synthesis and additive manufacturing ((**a**) Direct printing technique for the hybrids. (**b**) Paraffin mold. (**c**) Indirect printing method for the hybrids.), reprinted from Ref. [133].

In bone tissue engineering, scaffolds serve as a foundation for cells to adhere, multiply, differentiate, and mature into functional bone tissue [2]. It is widely acknowledged that the scaffold degradation kinetics should align with the new bone growth rate. Moreover, the scaffold design for tissue engineering of bone necessitates specific parameter fulfillment, including biodegradability, a high level of porosity with interconnected pores, appropriate mechanical characteristics, and osteoconductivity, in addition to the potential for commercialization [129]. As previously stated, BGs are commonly regarded as scaffold materials in the field of bone tissue engineering due to their exceptional bioactivity and ability to release ions, which ultimately promote osteoconductive and osteoinductive properties [130]. ZBG-based scaffolds are gaining more recognition due to the crucial zinc role in bone mineralization and formation. The methods used for producing ZBGs are summarized in Table 2, along with potential medical device forms for these types of bioactive glasses. It is important to note that most BGs, including zinc-doped BGs, are currently investigated in the form of granules or powders. However, there is significant hope that scaffolds and other forms will eventually reach the market. The human body primarily stores zinc in bone tissue, making it a crucial reservoir. Scientific investigations propose that the administration of zinc supplements enhances the expression of osteoprotegrin (OPG). Although the precise cellular and molecular mechanisms by which zinc facilitates bone growth remain unclear, it is established that zinc exerts beneficial effects on chondrocyte and osteoblast functions while suppressing osteoclastic bone resorption [131,132]. In a study conducted by Elahpour et al. [133], novel Zn-doped hybrid materials comprising polycaprolactone (PCL) in addition to BG were designed. The hybrid material consisted of 30 wt.% BG based on 75SiO_2_–15CaO–10ZnO (at%) and 70 wt.% PCL. This hybrid was produced using the sol-gel formation of BGs within a PCL solution. SiO_2_–CaO–ZnO BG/PCL hybrid scaffolds were then additively manufactured using fused deposition modeling (FDM) techniques, specifically indirect and direct 3D printing. The scaffold performance was assessed by evaluating physicochemical properties in addition to in vitro 3D cell culture. The results demonstrated great biocompatibility in addition to promising release kinetics. To further evaluate the potential of these hybrid scaffolds for bone tissue engineering applications, in vitro cell behavior was assessed using bone-marrow-derived human mesenchymal cells (hMSCs) via indirect and direct cell viability tests. These findings highlight the potential of hybrid scaffolds in bone tissue engineering. The primary morphological properties as observed by SEM of printed scaffolds are illustrated in Figure 8 through a schematic representation.

Haimi et al. [125] fabricated BG scaffolds utilizing fibers produced through melt spinning within a Na_2_O–K_2_O–MgO–CaO–B_2_O_3_–TiO_2_–P_2_O_5_–SiO_2_ system. Various ZnO compositions ranging from 0 to 5 mol% were utilized as substitutes for CaO. There was no evidence of crystallization, even in the scaffold containing the maximum ZnO content (5 mol%). The synthesized BG fibers containing zinc were used to create three-dimensional scaffolds measuring 14 × 14 × 5 mm^3^, with 70% porosity. The scaffolds were utilized in cell culture investigations. Human adipose stem cells (hASCs) exhibited good spreading across all the scaffolds and displayed an elongated phenotype. Qualitative live/dead staining indicated that the zinc inclusion hindered cell dispersing and growth. Nevertheless, there was no notable impact on ALP activity, DNA content, and osteopontin hASC concentration with zinc addition when assessed quantitatively. Wang et al. [134] successfully synthesized hierarchical microporous and mesoporous CaO–SiO_2_–P_2_O_5_ BG scaffolds doped with zinc (0.075, 0.15, 0.225 mole) through the sol-gel method. The green specimens were subjected to sintering at 700 °C with a 1 °C·min^−1^ heating rate to fabricate porous scaffolds. The resulting scaffolds exhibited open pores ranging from 100 to 400 µm in diameter, with a pore wall thickness varying between 10 and 50 µm. The response of mesenchymal stem cells (MSC) to the zinc-containing scaffolds was investigated. It was found that the zinc-doped scaffolds did not display any cytotoxic effects, and both MSC proliferation and ALP activity were enhanced by the slow release of zinc into the culture medium. The zinc concentration in the culture medium ranged from 0 to 0.59 ± 0.03 mg L^−1^, which was determined as non-toxic and it was anticipated this would stimulate bone growth in vivo.

**Figure 9:**
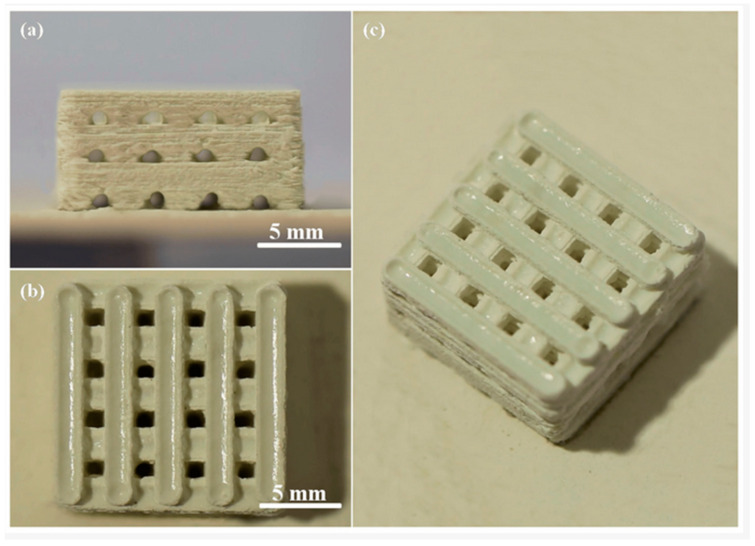
BG/CaSO_4_ scaffold, (**a**) a front view, (**b**) a top view, and (**c**) an oblique view, all incorporating zinc oxide whiskers (ZnOw), reprinted from Ref. [135].

Shuai et al. [135] utilized selective laser sintering (SLS) to fabricate porous CaSO_4_/BG scaffolds incorporated with zinc oxide whisker, ZnOw (Figure 9). They did not specify the exact composition of the bioactive glass, but it appears they studied 45S5 Bioglass. The dimensions of the cubic scaffolds were approximately 14 mm × 14 mm × 6.5 mm (length × width × height). These scaffolds exhibited interconnected pore channels, enabling the formation of a three-dimensional (3D) porous structure through orthogonal branching. The pore size and wall thickness were measured to be approximately 1.2 mm and 1.8 mm, respectively. Previous studies have highlighted the significance of a pore size exceeding 300 μm for facilitating new bone vascularization in addition to ingrowth within the constructs [135].

### 5.2. Bone Regeneration and Wound Healing

Zn has been found to possess anti-inflammatory and antibacterial properties, as well as an ability to inhibit the differentiation of osteoclasts [136,137]. Previous studies have also indicated the beneficial impact of zinc on the healing process of bone fractures [138]. Furthermore, zinc has been reported to stimulate angiogenesis, a crucial process for bone healing [139]. In 2015, zinc-containing borosilicate bioactive glass was synthesized by adding 1.5, 5, and 10 wt.% ZnO into the glass composition. The presence of Zn was found to slow down the glass degradation process in simulated body fluid without affecting hydroxyapatite formation. The ZBG-based scaffolds released Zn ions gradually for over 8 weeks and showed good compatibility with human bone marrow-derived stem cells, promoting their attachment and ALP activity. In an animal study on rat calvarial defects, the 5Zn-BG scaffolds outperformed undoped scaffolds in promoting bone tissue regeneration over an 8-week period. The study suggests that these ZBG scaffolds could be promising for applications in bone tissue repair and regeneration due to their enhanced osteogenic properties [140]. In 2019, Rajzer et al. [141] demonstrated the successful incorporation of ZBG and BG into fibrous membranes produced by electrospinning. ALP activity and mineralization were found to be greater in polycaprolactone membranes modified with ZBG compared to pure PCL membranes or control materials. The results indicated that the presence of Zn^2+^ ions in the electrospun membranes significantly impacted NHOst cell osteogenic differentiation.

**Figure 10:**
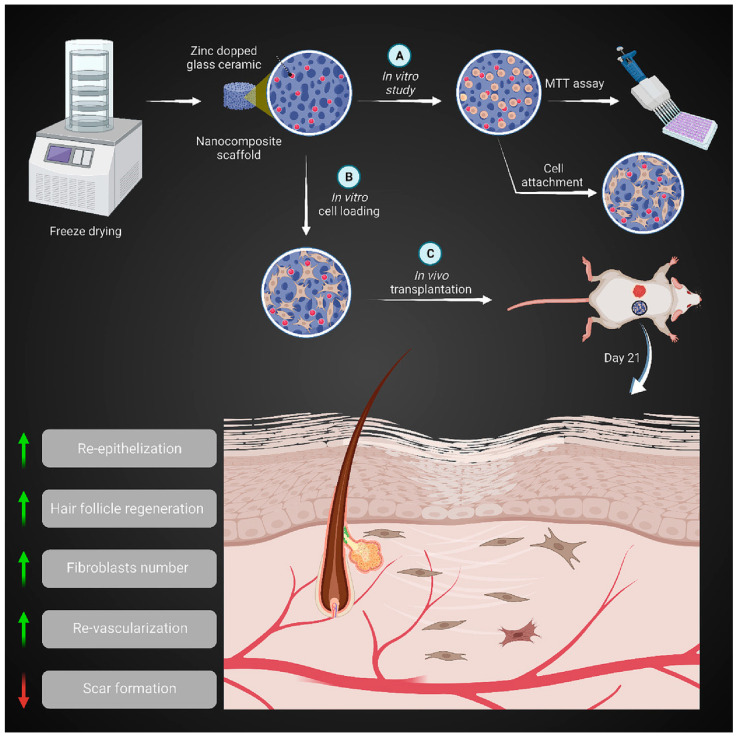
The bioactive nanocomposite scaffold of Col-Gel containing ZBGC nanoparticles loaded with mouse embryonic fibroblasts can be employed as a skin substitute to ameliorate cutaneous wound regeneration ((**A**) In vitro study (**B**) In vitro cell loading (**C**) In vivo transplantation), reprinted with permission from Ref. [144]. Copyright 2023 Elsevier.

Zinc-containing bioactive glass-ceramic (ZBGC) has been found to enhance biocompatibility and angiogenesis properties, as previously mentioned [72,142]. In such glass-ceramics, combeite (Na_6_Ca_3_Si_6_O_18_) and silicorhenanite (Na_2_Ca_4_(PO_4_)_2_SiO_4_) are partially crystallized, and crystalline and amorphous phases co-exist in the structure. In the Zn-doped BGC, a minor peak shift toward higher angles and a decrease in peak intensities were detected [143]. ZBGC was integrated into a porous scaffold made of gelatin (Gel) and collagen (Col). Findings revealed that the Col-Gel/ZBGC nanocomposite biodegradation rate was comparable to the skin tissue regeneration rate in vivo. Notably, the wound healing results at a macroscopic level demonstrated that the Col-Gel/ZBGC scaffold loaded with mouse embryonic fibroblast exhibited the smallest wound size, showing a faster healing process. Histopathological evaluations further confirmed the optimal wound regeneration in the Col-Gel/ZBGC nanocomposites loaded with mouse embryonic fibroblasts, as evidenced by angiogenesis and epithelialization. Additionally, there was an increase in the hair follicle and fibroblast number. Therefore, the bioactive nanocomposite scaffold consisting of Col-Gel and ZBGC nanoparticles loaded with mouse embryonic fibroblasts holds promise as an effective skin substitute for enhancing cutaneous wound repair [144]. The potential of nano-ZBGC in stimulating the regeneration of bone tissue was examined in a separate study conducted by Paramita et al. [145]. The incorporation of zinc into the ceramics not only improved their crystalline physiology but also enhanced their biological ability and antibacterial properties at the nanoparticle level. In vitro evaluations, including hemo-compatibility against mineral deposition works, biocompatibility, cytocompatibility, cytotoxicity, and hRBCs in mammalian cell lines, demonstrated the superior nano-ZBGC osteogenic effect compared to nano-BGC. The presence of bone-like hydroxyapatite in the nanoparticles further confirms their enhanced bone regenerative characteristics. Importantly, no toxic effects were observed, although higher concentrations and longer nanoparticle incubation periods did result in increased toxicity [145]. Numerous studies have demonstrated that ZBGs can boost angiogenesis and hasten wound repair by upregulating VEGF mRNA and other molecular mechanisms involving zinc metallothioneins and metalloenzymes [135,146,147]. In general, zinc-containing bioactive glass (ZBG) holds significant potential for skin repair and regeneration due to its various beneficial effects on wound-healing processes. As shown schematically in Figure 10, a ZBGC, when combined with gelatin (Gel) and collagen (Col), can contribute to skin regeneration.

### 5.3. Coatings of Orthopedic Implant

Zinc serves as a regional controller of bone and promotes bone metabolism both in laboratory settings and within living organisms [148]. Hence, zinc has been regarded as a dynamic elemental inclusion in coatings utilized for orthopedic purposes. Zinc’s unique properties can be utilized to create antimicrobial coatings for metallic implants. One example of incorporating zinc into the TiO_2_ coating sub-surface is through the utilization of plasma immersion ion implantation and deposition [149]. Previous studies revealed that rat bone marrow cells exhibited increased upregulated osteogenic-related genes and ALP activity when exposed to zinc-incorporated surfaces as opposed to zinc-free surfaces. Furthermore, zinc possesses antimicrobial properties [150]. Zinc was incorporated into mesoporous bioactive glass nanoparticles (MBGNs) to promote the activity of osteoblasts, augment bone formation, and improve antimicrobial properties. Lotfibakhshaiesh et al. [151] introduced 3 mol% ZnO into a glass system composed of SiO_2_–MgO–Na_2_O–K_2_O–ZnO–P_2_O_5_–CaO–SrO. The purpose of this addition was twofold: to hinder crystallization and to enable a controlled Zn^2+^ ions release from the glass. Primarily due to its bactericidal characteristics in addition to its importance in the treatment of wounds and the construction of bones, this material is highly valued [152]. The investigation of various BG compositions revealed a preference for sintering without crystallization, leading to a wider processing window. This was true for all compositions except when strontium was used as a complete replacement for calcium. BG coatings were successfully created with a thermal expansion coefficient similar to Ti-6Al-4V alloy. Limited research has been conducted on coatings made from ZBGs according to the available literature. Future studies should explore the antibacterial properties of zinc by designing innovative ZBG-based coatings. Similarly, the application of a nanoparticulate bioactive glass surface on porous titanium implants enhances osteointegration and accelerates bone generation within the implant pores compared to implants without a coating. Also, the enhancement of mechanical properties in the coating on a Ti-6Al-4V substrate is achieved through the addition of nanosized hydroxyapatite (HAp) into a BG coating containing ZnO. This incorporation of nanosized HAp does not hinder the in vitro bioactivity of the coating [153].

**Figure 11:**
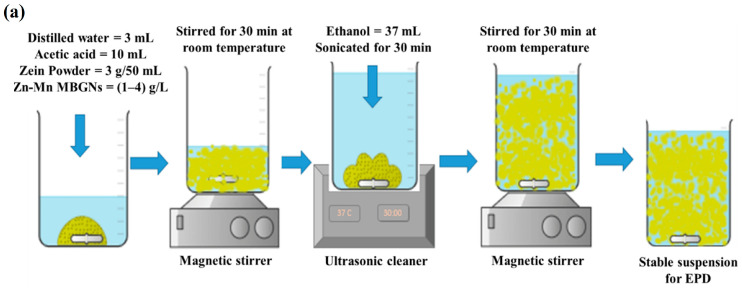

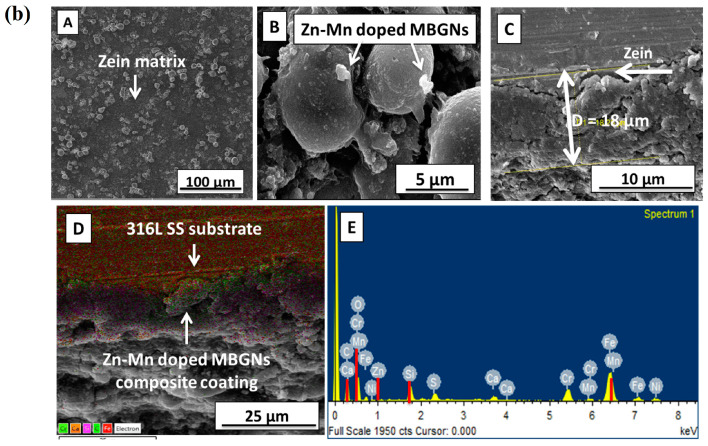
(**a**) Preparation process of a stable zein/Zn-Mn MBGN suspension. (**b**) SEM images were taken of zein/Zn-Mn MBGN coatings. Images captured different aspects of the coating: (**A**) a low magnification image revealed a uniform deposition of the coating, (**B**) a high magnification image showed the distribution of Zn-Mn MBGNs on zein particles, (**C**) a cross-section image provided a view of the coating’s internal structure, (**D**) elemental mapping was conducted to analyze the elemental distribution within the coating, and (**E**) an energy-dispersive spectroscopy (EDS) of the composite coating was obtained to identify the elements present, reprinted from Ref. [31].

In a study conducted by Chen et al. [154], a mesoporous bioactive glass (MBG) was successfully applied to porous titanium (Ti) scaffolds using a pulling and dipping method. However, the BG coating exhibited severe cracking. By introducing zinc into the MBG coating, a significant reduction in the specific pore diameter, surface area, and pore volume was achieved. The Zn ions played a crucial role in enhancing the structural integrity of the glass, leading to a substantial decrease in the overall ability of hydroxyapatite formation in the ZMBG scaffolds. In vitro antibacterial experiments demonstrated that Zn exhibited a certain level of antibacterial effect, which was directly proportional to the Zn content. Furthermore, the continuous release of Zn ions in a phosphate-buffered saline (PBS) solution over a period of 28 days exhibited a long-term bactericidal effect. Moreover, the Zn scaffolds were found to stimulate the proliferation and adhesion of MC3T3-E1 cells. Interestingly, as the Zn amount added increased, the coated samples exhibited enhanced antibacterial properties but reduced cytocompatibility. This is of significant importance in various applications where inhibiting bacterial growth is crucial, such as in the development of implant coatings. In a study conducted by Batool et al. [31], a composite coating composed of MBGNs and zein, a biopolymer found in corn, was developed for orthopedic purposes. The simultaneous deposition of Zn–Mn-containing MBGN and zein through electrophoretic deposition (EPD) was examined. The MBG structure aids in enhancing osteo-induction by offering a large active surface area for osteoblast cells to adhere to and grow. Zinc is incorporated for its antibacterial properties and manganese for its osteogenic effects. Additionally, the Taguchi design of experiments (DoE) was utilized to optimize zein/Zn–Mn MBGN coatings. This approach will assist in identifying the key factors (such as voltage, time, and concentration) that influence the quality of deposits, thereby enhancing the understanding of the deposition kinetics of zein-based composite coatings. Figure 11a illustrates the sequential stages involved in the preparation process of a stable zein/Zn-Mn MBGN suspension, which is subsequently utilized for depositing a composite coating onto 316L stainless steel. The optimized EPD parameters determined through the Taguchi DoE method were utilized in the preparation of the coatings. Initially, an optical microscope was employed to examine the coatings, revealing a consistent coverage of the 316L SS substrate by the zein/Zn–Mn MBGN coating (Figure 11b).

### 5.4. Bone Grafts and Filling Materials

The primary property of bone graft materials, whether autografts, allografts, xenografts, or synthetic engineered/biomaterial-based substitutes, is osteoconductive, which promotes bone formation on the material surface [145]. Some bone grafts also possess additional properties which may have significant positive impacts on bone regeneration. As advanced materials, these specially featured bone grafts actively aid in bone formation and can cause faster and more effective healing responses. Bioactive glasses have received considerable attention for their unique bone formation properties [155]. These materials serve as frameworks for new bone growth, aiding in restoring bone volume for successful dental implant treatment [156]. The choice of bone-filling material depends on factors like the patient’s condition, the need for additional procedures, and the desired outcome of the grafting procedure [157].

Oh et al. [107] explored the effects of adding trace amounts of zinc to BG granules produced via the sol-gel technique on mesenchymal stem cell (MSC) proliferation and bone-forming potential. The BG granules were formulated with varying compositions (70SiO_2_–(30 − *x*)CaO–*x*ZnO in mol%, *x* = 2 and 5). In vitro experiments in SBF showed that the granules’ surfaces were completely coated with a thick layer of apatite minerals after 7 days. ALP activity, an osteogenic differentiation marker, was used to assess the impact of ZBG granules on MSC differentiation, revealing that the presence of zinc prompted MSCs to differentiate to a greater extent compared to the zinc-free granules. The study concluded that the addition of zinc to the BG granules not only supported adult stem cell growth but also stimulated their further differentiation into the osteogenic lineage. Boyd et al. [158] conducted a comprehensive investigation on the physical and structural characteristics and in vitro cytotoxicity, in addition to bone graft in vivo biocompatibility made from calcium–strontium–zinc–silicate glass. One of the glass systems that was prepared showed the most favorable behavior in vitro among the various options. This particular graft had a composition of 0.28 mol fraction SrO, 0.32 mol fraction ZnO, and 0.40 mol fraction SiO_2_. It induced only minimal cytotoxic effects on L929 mouse fibroblast cells, with an up to 95% cell viability rate. In comparison, the commercially available bone graft, Novaboner, showed a 72% lower cell viability rate. Furthermore, when tested in rats, the developed grafts performed equally well in both healthy and osteoporotic tissue. Bone grafts in addition to bone filling materials in dentistry play a crucial role in promoting bone formation.

Periodontal diseases and dental caries are the two most prevalent chronic illnesses observed in humans [159]. Uskoković et al. [160] reported on a novel nanocomposite material fabrication and characterization for dental applications. The material is composed of Na_2_O–CaO–P_2_O_5_–SiO_2_ bioactive glass-ceramic nanoparticles doped with zinc and niobium and hybridized with chitosan. The addition of zinc ions inhibited partial recrystallization during annealing by interfering with Si–O network restructuring, proportional to its concentration. The electrostatic attraction between the chitosan aminated hydrocarbon chains and silica due to the negatively charged silanol groups may interact with decalcified dentin collagen fibrils, making the material potentially useful for adhesive fillings of carious lesions. Both the doped and undoped bioactive glass-ceramics interacted with odontoblast-like cells, highlighting the desirability of further research into their application in minimally invasive reparative dentistry [160].

**Table 2:** Applications of Zinc-Containing Bioactive Glasses.

BG Composition	Zinc Content	Method	Applications	Medical Device Form	Main Results	Ref.
Zn-doped 45S5-derived systems	5, 20 mol%	Melt-quenching	Tissue repair	Powder	Zinc addition to bioactive 45S5 glass reduced the overall leaching activity and inhibited hydroxo-carbonate apatite formation at high Zn concentrations Only the 5 wt.% Zn-containing glass showed reduced solubility, bioactivity, and conditions that allowed endothelial cell growth	[126]
xZnO–(57.0 − *x*)CaO–35.4SiO_2_–7.2P_2_O_5_–0.4CaF_2_ in mol%	0–14.2 mol%	Melt-quenching	Bone formation	Powder	Addition of ZnO increased the chemical durability of the glass-ceramics, resulting in a slower rate of apatite formation The release of zinc from the glass-ceramics increased with higher ZnO content	[161]
SrO–ZnO–SiO_2_	0.32 (mol fraction)	Melt-quenching	Bone graft	Granule	In cell culture experiments, these glasses showed mild to moderate cytopathic effects on L929 mouse fibroblast cells	[158]
Na_2_O–K_2_O–MgO–Cao–P_2_O_5_–B_2_O_3_–TiO_2_–SiO_2_–ZnO	0–5 mol%	Melt-spinning	Bone tissue engineering	Scaffold	Without impact on hASC proliferation and osteogenesis, S. mutans, DNA content	[125]
SiO_2_–CaO–P_2_O_5_–ZnO	1, 3, 5 mol%	Sol-gel	Bone integration	powder	Zinc accelerates bone cell differentiation. Apatite layer formed on all glass powders when immersed in SBF	[162]
SiO_2_–ZnO–CaO–SrO–Na_2_O	0.2–0 mole (fraction)	Melt-quenching	Osseous integration	Scaffold	Efficient antibacterial characteristics Stimulation of osteoblast differentiation Prevention of osteoclast resorption	[163]
SiO_2_–CaO– P_2_O_5_–ZnO	0, 4, 7 mol%	Evaporation-induced self-assembly	Bone regeneration	Scaffold	4% exhibited a higher release of zinc ions (Zn^2+^) Showed a 3.6-fold higher ability to inhibit the growth of *S. aureus*.	[127]
SiO_2_–Na_2_O–ZnO–CaO–MgO–P_2_O_5_	1.24 and 2.4 mol%	Melt-quenching	Wound healing Orthopedic infections	Scaffold	Scaffolds demonstrate potential effectiveness for orthopedic infections and bone void filling. ZBG requires thorough investigation to fully understand its cytotoxicity and wound healing properties	[164]
ZnO–Na_2_O–SiO_2_–CaO–P_2_O_5_–MgO	6, 8, 10 mol%	Sol-gel	Bone regeneration	Powder	The formation of a hydroxyl apatite layer Drug release properties exhibited by the samples	[165]
SiO_2_–CaO–ZnO	2 and 5 mol%	Sol-gel	Odontogenic and angiogenic	Powder	Demonstrate the activation of odontogenic differentiation and the facilitation of angiogenesis in cell cultures when ZBG is integrated within CPCs.	[116]
B_2_O_3_–MgO–SiO_2_–Na_2_O–CaO–P_2_O_5_–ZnO	2 mol%	Sol-gel	Bone regeneration	Powder	All the samples prepared were found to be non-toxic, with over 70.3% viable cells observed. Attachment with MG63 cell line demonstrated that the samples created a favorable environment for cell line growth	[166]
49CaO–5ZnO–6P_2_O_5_–40SiO_2_	5 mol%	Sol-gel and electrospinning	Nasal tissues treatment	Scaffold	Producing fibrous membranes with Zn-doped BG integrated Impacting the microstructure of PCL membranes Redeciding apatite precipitation by Zn ions Leading to higher mineralization and ALP activity Influencing the osteogenic differentiation of NHOst cells Providing antibacterial properties in nasal implants	[141]
70SiO_2_–25CaO–5ZnO	8 mol%	Microemulsion assisted sol-gel	Bone regeneration Wound healing	Powders	Releasing Zn^2+^ ions in low concentration Promising for applications in both hard and soft tissue engineering Increasing the differentiation of osteoblast-like cells (MG-63) by ZBGNs	[43]
(I) ZnO–SiO_2_–P_2_O_5_–CaO (II) Ag_2_O–SiO_2_–P_2_O_5_–CaO	2 and 4 mol%	Sol-gel	Bone tissue	Powder	Antibacterial investigations revealed that Ag-doped samples had a better antibacterial effect compared to Zn-doped ones.	[93]
80SiO_2_–15CaO–5P_2_O_5_–4ZnO	4 mol%	Evaporation induced self-assembly	Bone infection	Scaffold	Increasing the antibacterial effect of the system by ZBGs Effect of zinc with antibiotics LEVO, VANCO, RIFAM, or GENTA. ZBGs showed antibacterial a synergistic antibacterial effect with LEVO and VANCO against *S. aureus* and with LEVO and GENTA against *E. coli* cultures.	[167]
*x*ZnO–22Na_2_O–24CaO–(54 − *x*)–P_2_O_5_	2, 4, 6, 8, 10 mol%	Melt-quenching	Bone implant	Powder	The glass transition temperature (T_g_) showed an increase with increasing ZnO content up to 8 mol% and then decreased for 10 mol% of ZnO. Vickers microhardness and toughness values of the BG increased with the rise in ZnO content due to the expansion of the glass network and the increase in inter-atomic spacing.	[80]
60SiO_2_–(36 − *x*)CaO–4P_2_O_5_–xZnO	1, 5 mol%	Sol-gel	Bone substitute An additive in toothpaste	Powder	The addition of ZnO to glass systems results in enhanced bioactivity and biocompatibility.	[168]
60SiO_2_–4P_2_O_5_–31CaO–*x*SrO– (5 − *x*)ZnO	1–5 mol%	Sol-gel	Bone substitute	Powder	The samples with 1% and 2% zinc showed enhanced viability as the immersion time increased up to day 7. Resulting in antibacterial activity in the vicinity of the bioactive glass.	[169]
SiO_2_ –CaO–P_2_O_5_–ZnO	1, 3, 5 mol%	Evaporation induced self-assembly	Coating materials	Scaffold	Increasing content of Zn from 1 to 5 mol%, resulted in a decline in apatite forming ability. Improving the antibacterial properties of the coatings by the addition of zinc Improving cell proliferation and exhibiting good cytocompatibility using MC3T3-E1 cells	[154]

## 6. Conclusions and Future Perspectives

Utilizing glass as a medium to regulate the release of zinc and enhance healing and antibacterial effects has shown great promise. While numerous studies have demonstrated the positive impact of zinc on healing in both soft and hard tissues, further research is needed to explore original glass compositions with varying zinc concentrations and release kinetics. Incorporating zinc into a glass network could allow control of zinc concentration and release rates. Additionally, understanding the dissolution mechanisms of glasses in different healing environments is crucial for targeting specific healing areas. The therapeutic potential of bioactive glasses can be augmented by adjusting their physicochemical properties. For example, zinc is known for its significant biological effects, such as boosting cell proliferation, promoting angiogenesis, and inhibiting bacterial growth. It is essential to maintain proper cell functionality while developing zinc-containing glasses with anti-inflammatory, antibacterial, or other beneficial properties. The future of therapeutic ions in glass depends on creating compositions that elicit reliable and beneficial biological responses. We have reviewed the progress made in the field of biomedical applications of bioactive glass containing zinc over the course of the last few years. Zinc-containing BGs, as detailed in this paper, have a profound effect on the overall performance of bioglasses, affecting their structure, physical properties, and biological activity. The addition of Zn was found to enhance the bioactive properties of the glasses, including tailored apatite-forming ability and in vitro cellular responses. The role of Zn in specific bioactive glass compositions can vary depending on its concentration, acting as either a network modifier or a glass former oxide. Until now, the percentage of zinc in zinc-doped bioactive glasses has ranged from trace amounts to as high as 20 mol%. The findings highlight the potential of Zn-containing bioactive glasses for various tissue engineering and regenerative medicine, particularly in the field of bone repair and regeneration. The enhanced bioactive performance of these glasses can be attributed to the favorable effects of Zn^2+^ ions, which are known to stimulate osteogenic differentiation and angiogenesis.

Future research should focus on elucidating the specific mechanisms by which Zn influences these biological processes. Additionally, the exploration of novel zinc-doped bioactive glass compositions, including the use of alternative network modifiers and the incorporation of other therapeutic ions, may lead to the development of even more versatile and effective biomaterials. Exploring the potential of multifunctional zinc-doped bioactive glasses to enhance bone regeneration, support angiogenesis, and combat infections represents a promising avenue of research.

## Figures and Tables

**Table 1:** The comparison between the sol-gel and melt-quenching methods.

Method	Advantages	Drawbacks	Ref.
**Sol-gel**	Easy processing Product monotony Lower operating temperature Synthesis of pure substances	Expensive and toxic raw ingredients Longer and more complex process	[59,68,69]
**Melt-quenching**	Mass production is made possible Low-cost and Simple method	Compositional limitation High-temperature processing The inability to accurately control the qualities of the final product (e.g., obtaining particles with uniform size distribution and regular shape)	[70,71,72,73,74]

## Data Availability

No data were used for the research described in the article.
